# Supplementary material for: The interactome of intact mitochondria by cross-linking mass spectrometry provides evidence for coexisting respiratory supercomplexes
Source: Mol Cell Proteomics. 2017 Dec 8;17(2):216–32. doi: 10.1074/mcp.RA117.000470 (PMC5795388; doi:10.1074/mcp.RA117.000470)
Supplement: Supplemental Data [file supp_RA117.000470_133922_0_supp_23978_fzffwf.zip › spectra_annotation/mito_spectra_annotation/310-1-11-1-7-1.pdf]

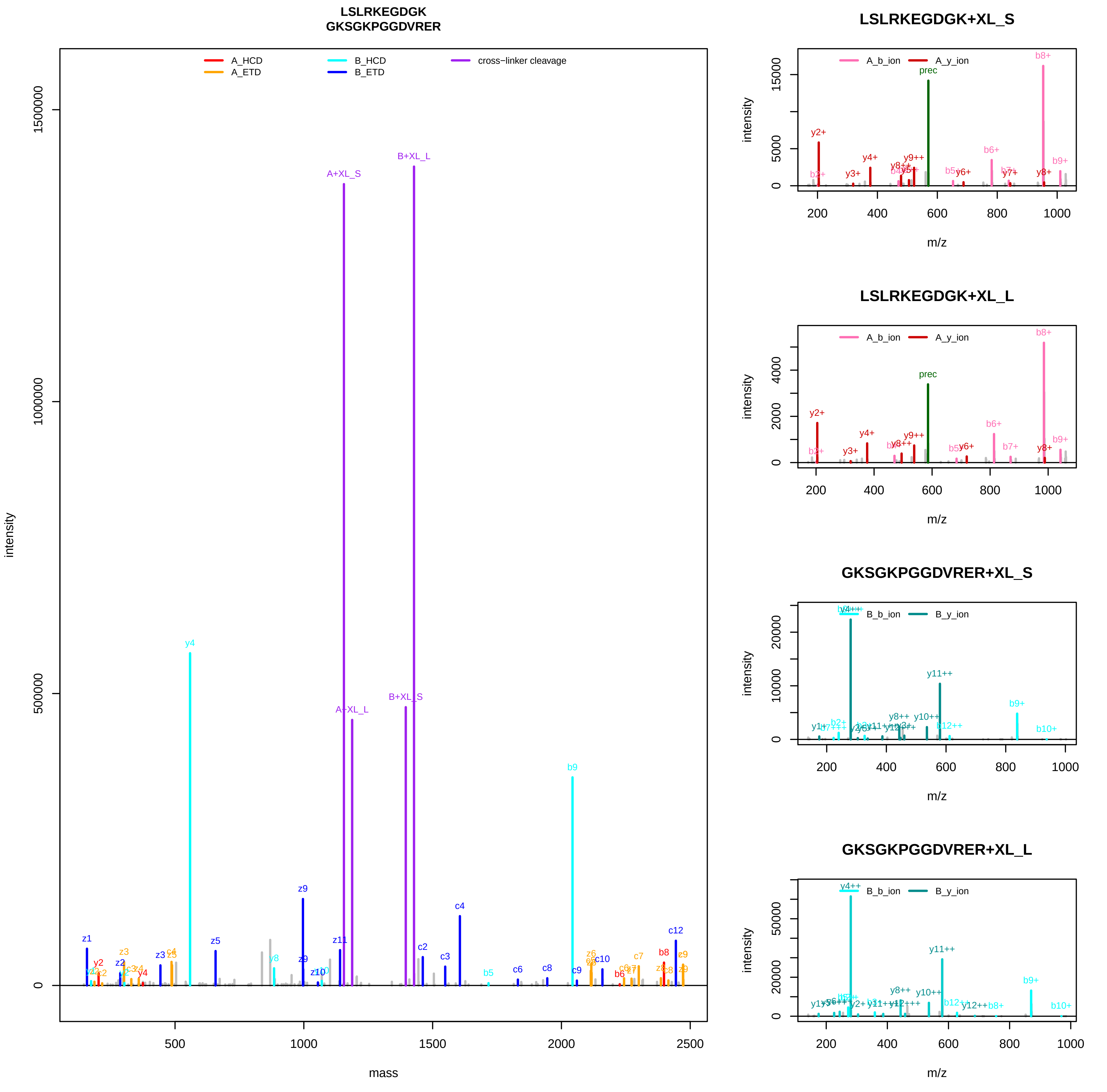
LSLRKEGDGK
GKSGKPGGDVRER
A_HCD
A_ETD
B_HCD
B_ETD
cross−linker cleavage
intensity
mass
0
500000
1000000
1500000
500
1000
1500
2000
2500
A+XL_S
B+XL_L
A+XL_L
B+XL_S
y4
b9
z9
c4
c12
z1
z5
z11
c2
c3
c6
c8
c9
c10
b5
b6
b8
c7
y8
LSLRKEGDGK+XL_S
LSLRKEGDGK+XL_L
GKSGKPGGDVRER+XL_S
GKSGKPGGDVRER+XL_L
A_b_ion
A_y_ion
B_b_ion
B_y_ion
prec
b8+
y2+
y4+
b6+
b9+
y8+
y9++
y11++
b10+
m/z
